# Supplementary material for: Large dynamics of a phase separating arginine-glycine-rich domain revealed via nuclear and electron spins
Source: Nat Commun. 2024 Feb 21;15:1610. doi: 10.1038/s41467-024-45788-w (PMC10881997; doi:10.1038/s41467-024-45788-w)
Supplement: Supplementary file 1 — Supplementary Information [file 41467_2024_45788_MOESM1_ESM.pdf]

Supplementary Information

Large dynamics of a phase separating arginine-glycine-rich domain revealed via nuclear and electron spins

Giuseppe Sicoli,[a‡] Daniel Sieme,[b‡] Kerstin Overkamp,[b] Mahdi Khalil,[a] Robin Backer,[c] Christian Griesinger,[b] Dieter Willbold,[c,d] Nasrollah Rezaei-Ghaleh[c,d]*

[a]CNRS UMR 8516, University of Lille, LASIRE, C4 Building, Avenue Paul Langevin, F–59655 Villeneuve d'Ascq, France.

[b]Department of NMR-based Structural Biology, Max Planck Institute for Multidisciplinary Sciences, Am Faßberg 11, D-37077 Göttingen, Germany.

[c]Heinrich Heine University (HHU) Düsseldorf, Faculty of Mathematics and Natural Sciences, Institute of Physical Biology, Universitätsstrasse 1, D-40225 Düsseldorf, Germany.

[d]Institute of Biological Information Processing, IBI-7: Structural Biochemistry, Forschungszentrum Jülich, Wilhelm-Johnen-Straße, D-52428 Jülich, Germany.

‡ These authors contributed equally.

*To whom correspondence may be addressed: Dr. Nasrollah Rezaei-Ghaleh

Email: Nasrollah.Rezaie.Ghaleh@hhu.de

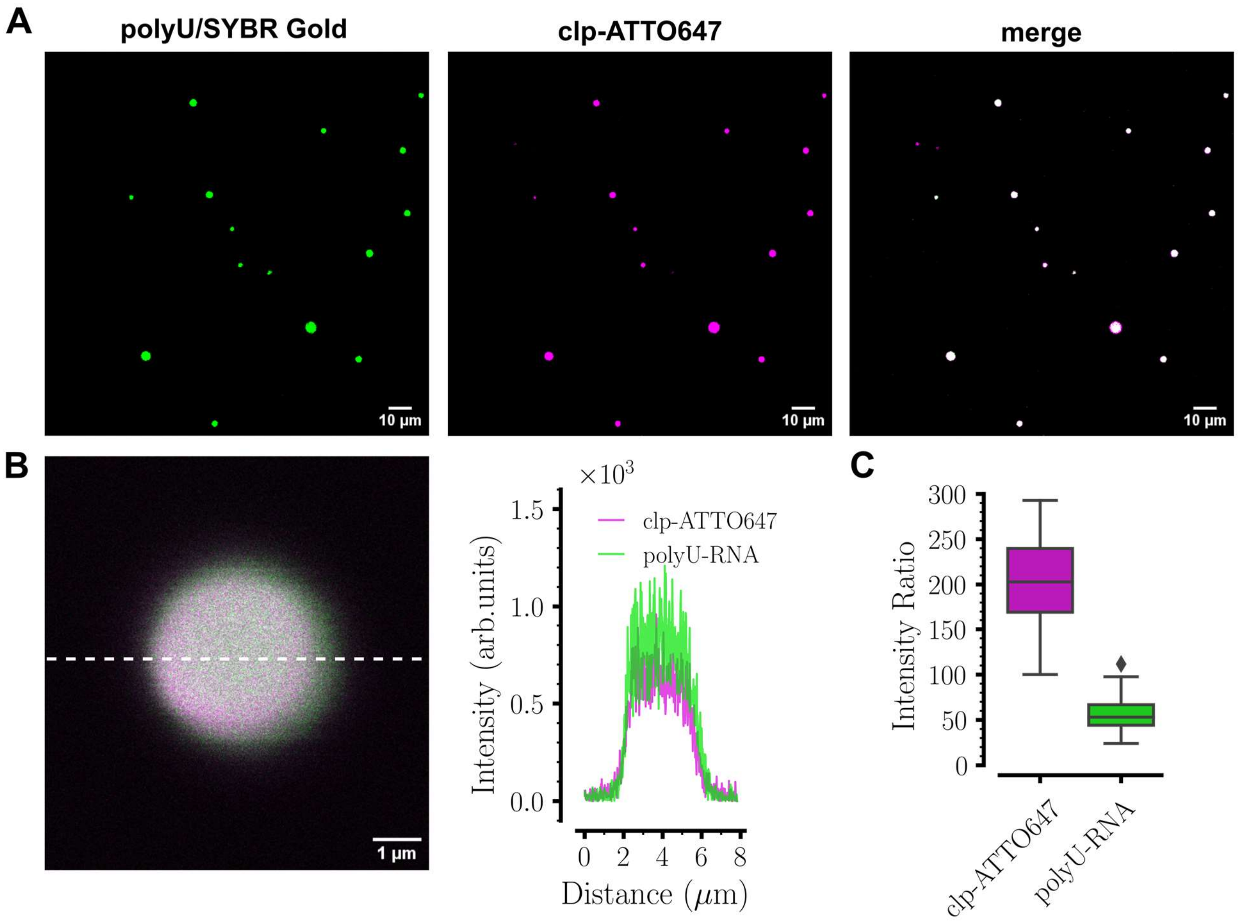

Supplementary Figure 1

Co-localization of polyU RNA (green) and ATTO647-coilin peptide (violet). **(A)** Confocal images of polyU RNA via SYBR Gold fluorescence (left), coilin peptide via ATTO647 fluorescence (middle) and the merged image showing the co-localization (right). **(B)** Line scan across a typical condensate, showing the fluorescence profile inside and outside of the droplet. **(C)** Boxplots showing the intensity ratios (IR) of coilin peptide and polyU RNA based on the mean intensity inside and outside of condensates. The boxes represent the interquartile range (IQR) which is between the 25th and the 75th percentile of the percentages of the respective intensity ratio. The whiskers represent 1.5 times the IQR. The black lines inside the boxes represent the medians. We analyzed all the droplets selected according to an un-biased threshold setting method. The number of analyzed droplets (n) was 65 for the coilin peptide and 85 for polyU RNA. Source data are provided as a Source Data file.

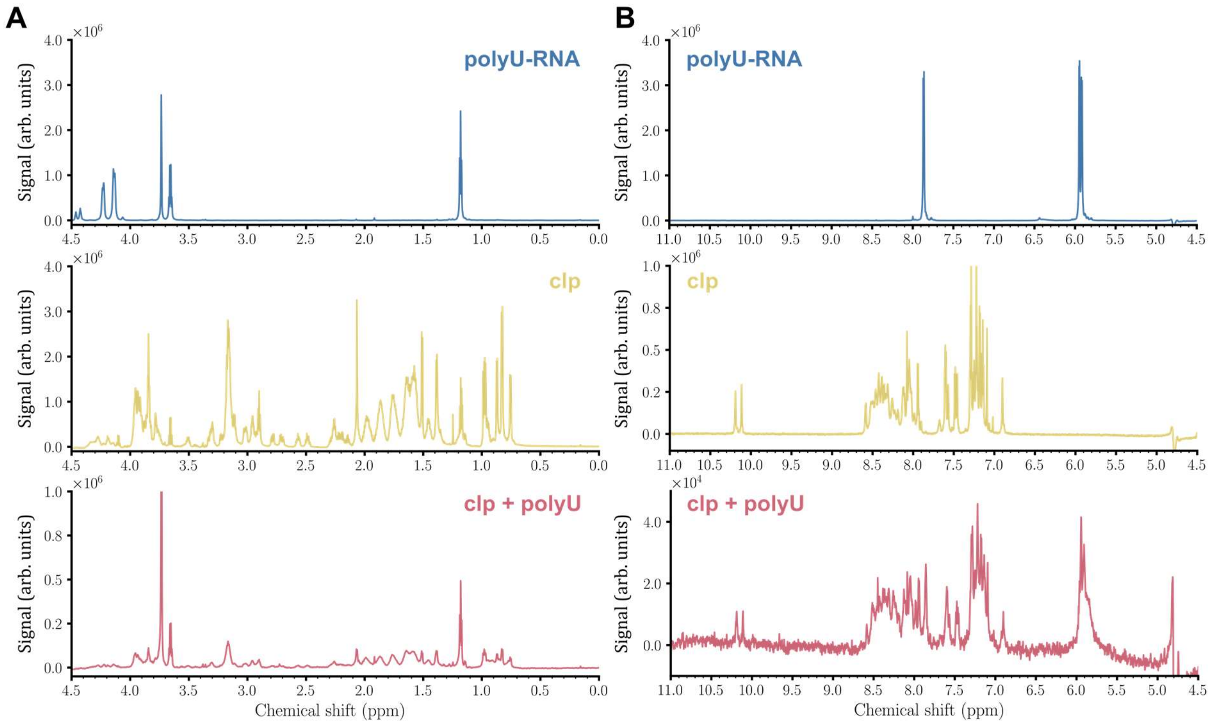

Supplementary Figure 2
1D ^{1}H NMR spectra of polyU RNA (blue, top), clp (gold, middle), and after mixing both (red, bottom), shown in two different regions of 0-4.5 ppm (left panel, **A**) and 4.5-11 ppm (right panel, **B**).

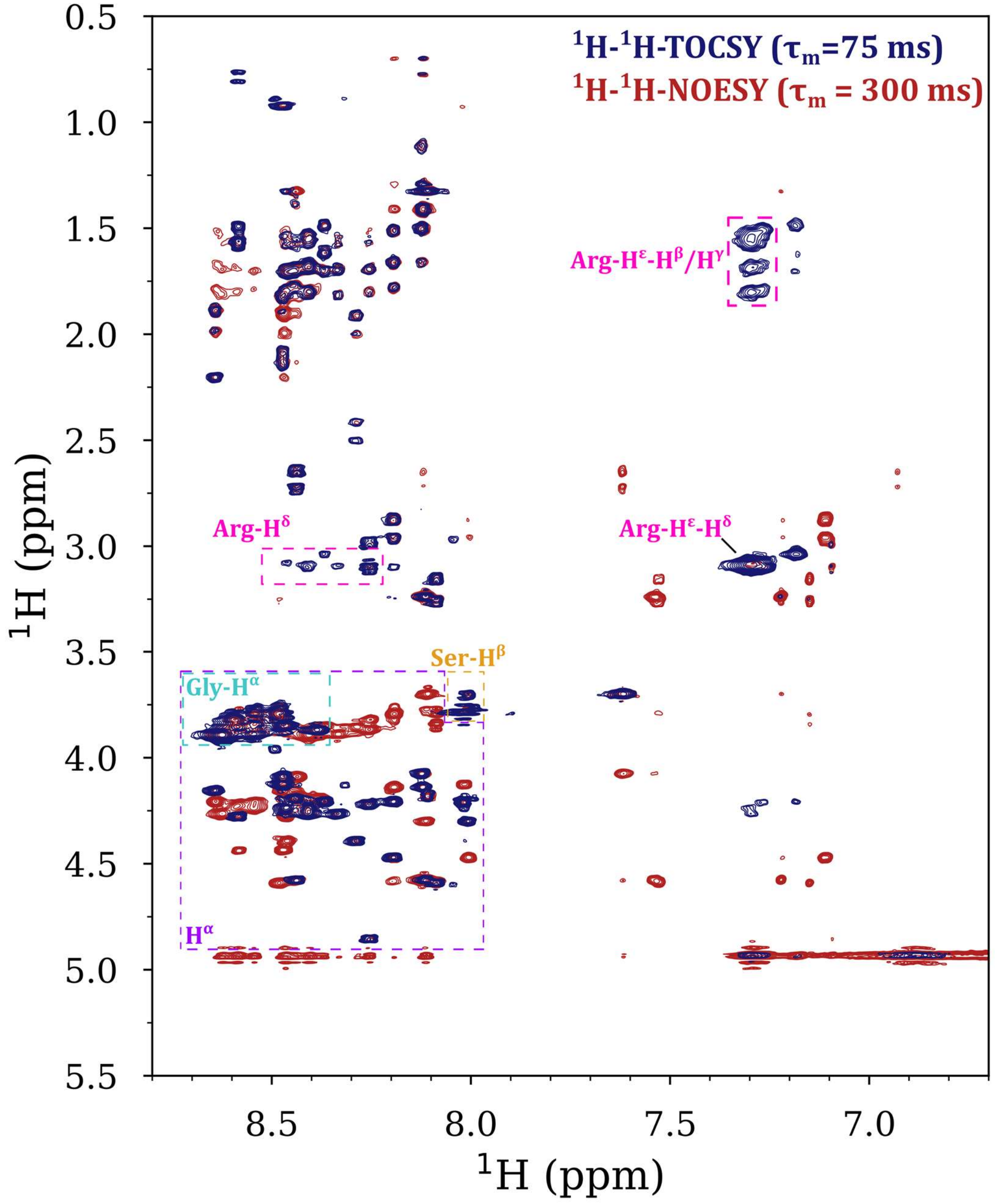

Supplementary Figure 3

2D ^{1}H,^{1}H TOCSY (blue) and NOESY (red) NMR spectra of coilin peptide (clp) used for obtaining the resonance assignment. The characteristic regions for glycine H^{α} and arginine H^{δ}, H^{ε}, H^{β}/H^{γ} and serine H^{β} correlations are highlighted at the corresponding positions in the spectra.

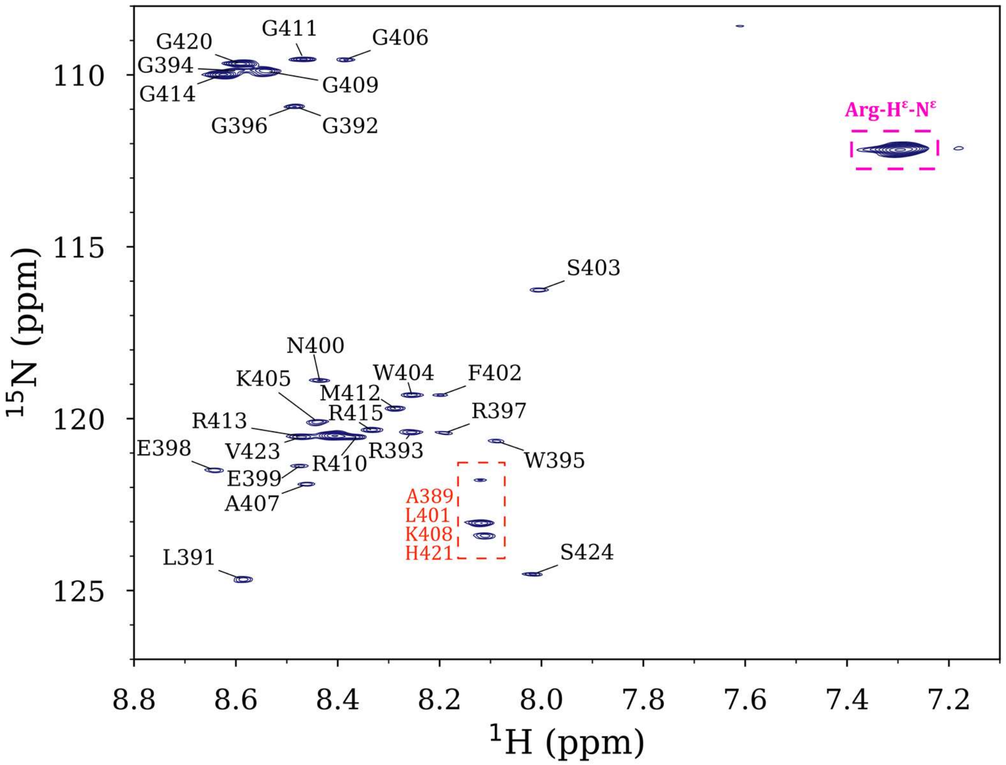

Supplementary Figure 4

2D natural abundance ^{15}N,^{1}H HSQC spectrum of clp. The resonance assignment was propagated from the 2D ^{1}H-^{1}H TOCSY and NOESY based on the assigned $^{1}H^{N}$ chemical shifts. The residues A389, L401, K408 and H421 (labeled red) could not be unambiguously assigned to the HSQC peaks based on their $^{1}H^{N}$ chemical shifts. The H^{ε}-N^{ε} correlation peaks of arginine sidechains appear as folded peaks at 112.2 ppm (original chemical shift: 84.2 ppm).

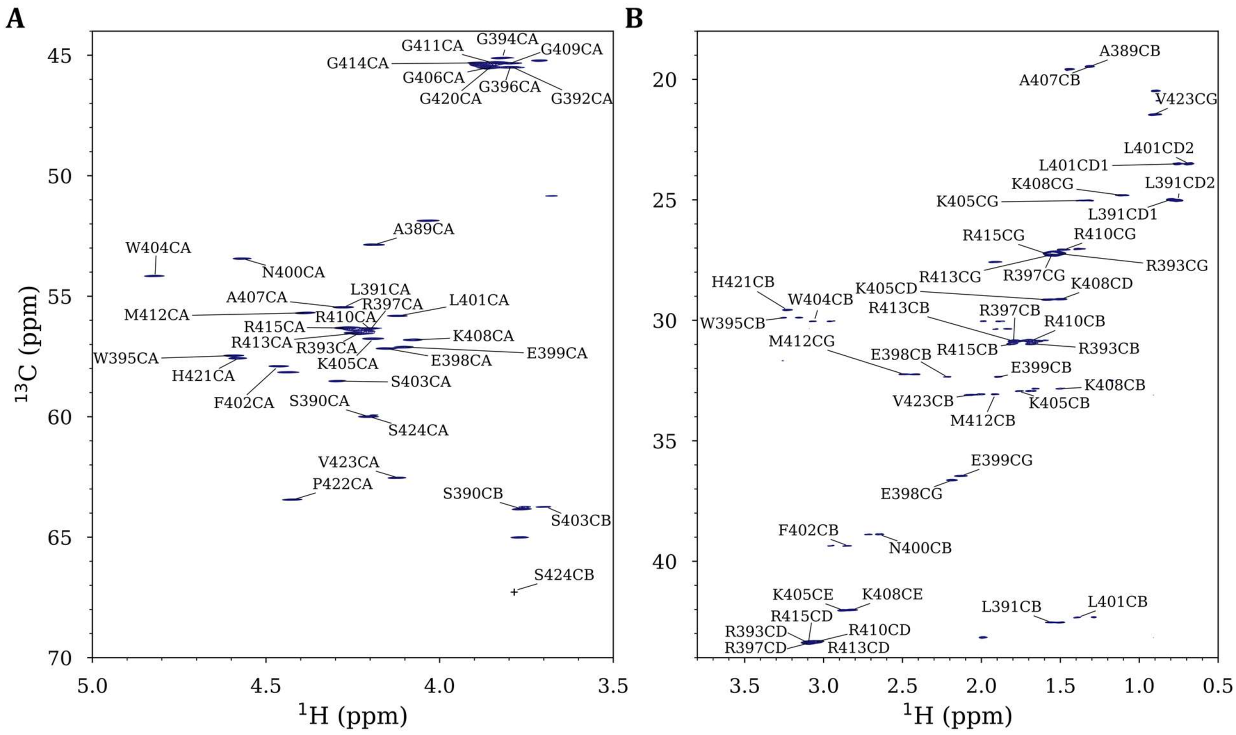

Supplementary Figure 5

Annotated 2D natural abundance ^{13}C,^{1}H HSQC spectrum of clp. **(A)** Spectral region above 44 ppm containing all of the C$^{\alpha}$ resonances and the C$^{\beta}$ resonances of the serine residues. The S424CB correlation peak (shown by a "+" sign) has low intensity and is not visible at the chosen contour level. **(B)** Spectral region below 44 ppm containing the remaining sidechain resonances of clp.

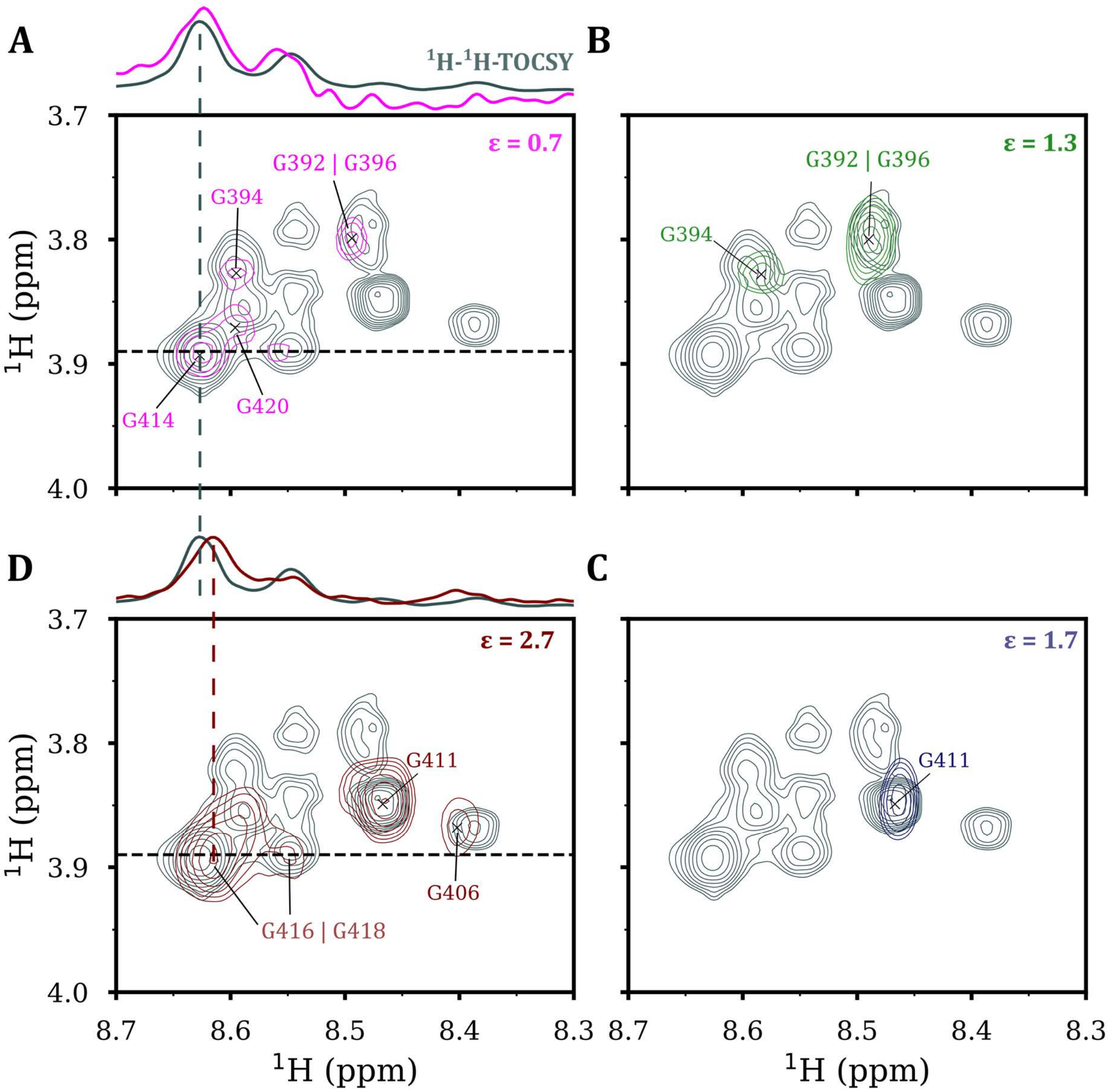

Supplementary Figure 6

Singlet-filtered TOCSY (sf-TOCSY) spectra of coilin peptide used for the assignment of glycine-based singlet-states. The sf-TOCSY spectra are superimposed onto the standard TOCSY spectra. Glycine-based singlet-states with various HA-HA Δν/J ratios (groups I-IV, **A-D**) were accessed (semi-)selectively in the indirect TOCSY dimension using experimental parameters reported in Supplementary Table 1 and correlated with HN resonances in the direct TOCSY dimension. The appearance of peaks in more than one spectrum is either due to peak overlap between glycines with different Δν/J ratios (suggested by small chemical shift differences between standard and sf-TOCSY peaks, dashed lines), or an imperfect selection potentially caused by the additional HN-HA coupling in the samples used for sf-TOCSY measurements (10%/90% D_2O/H_2O) when compared with the samples of 1D singlet-filtered measurements (in 100% D_2O).

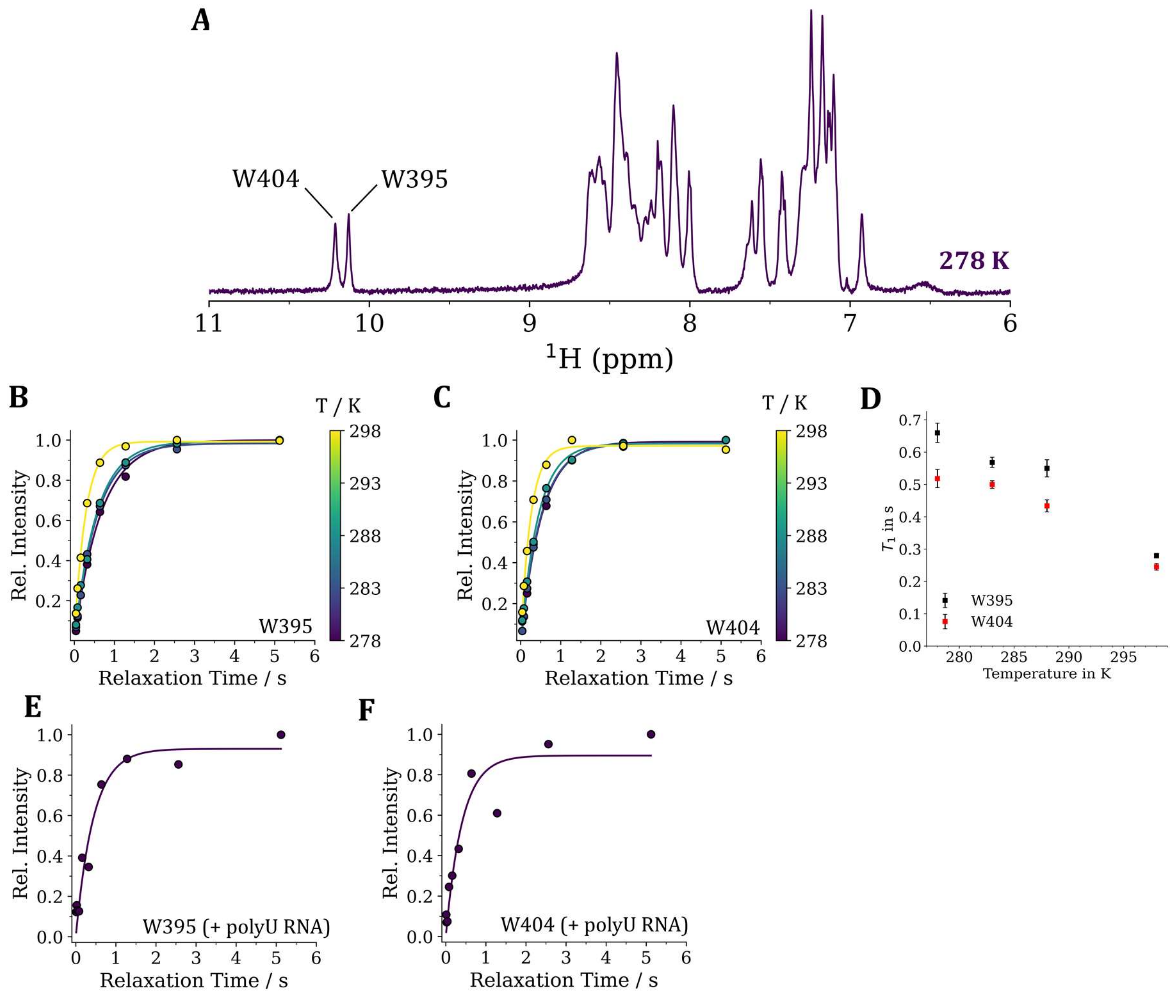

Supplementary Figure 7

Tryptophan side-chain dynamics probed via T_1 relaxation of H^{ε} protons. **(A)** 1D ^{1}H NMR spectrum of clp, showing the well-resolved H^{ε} resonances of W395 and W404. **(B,C)** Monoexponential non-linear least-squares fits for W395 and W404 sidechain H^{ε} resonances in the indicated temperature range. **(D)** T_1 relaxation times of W395 and W404 sidechain H^{ε} resonances in dependence of temperature. **(E,F)** Monoexponential non-linear least-squares fits for W395 and W404 sidechain H^{ε} resonances in presence of polyU RNA at 298 K. The obtained T_1 relaxation times of W395 and W404 in the presence of polyU RNA are reported in Fig. 4e in the main text. Source data are provided as a Source Data file.

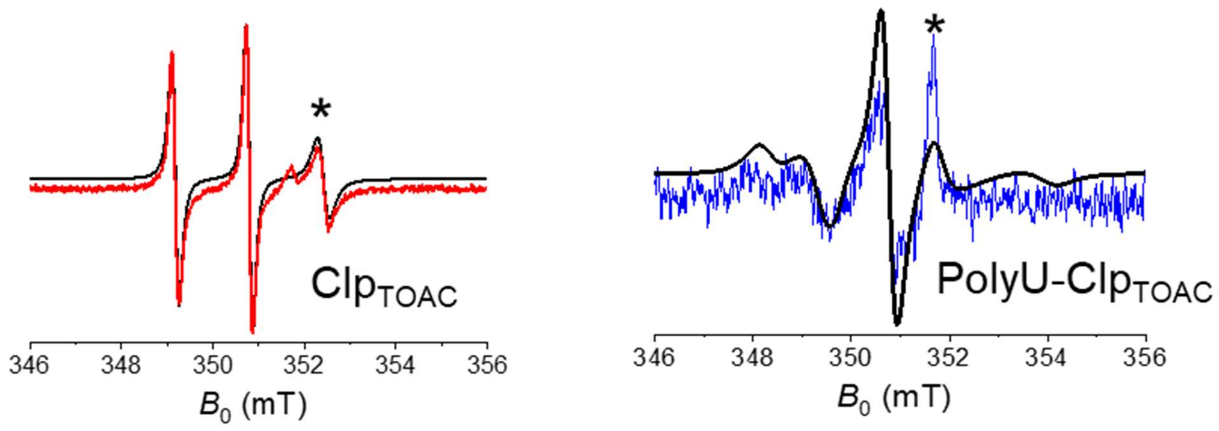

Supplementary Figure 8

CW EPR spectra on TOAC-labeled Clp without and with PolyU; the easyspin (version 5.2.35) fitting (https://easyspin.org/ , https://easyspin.org/easyspin/documentation/references.html) shows the different ranges of hyperfine coupling and rotational correlation times. (*) background-resonator signal. Source data are provided as a Source Data file.

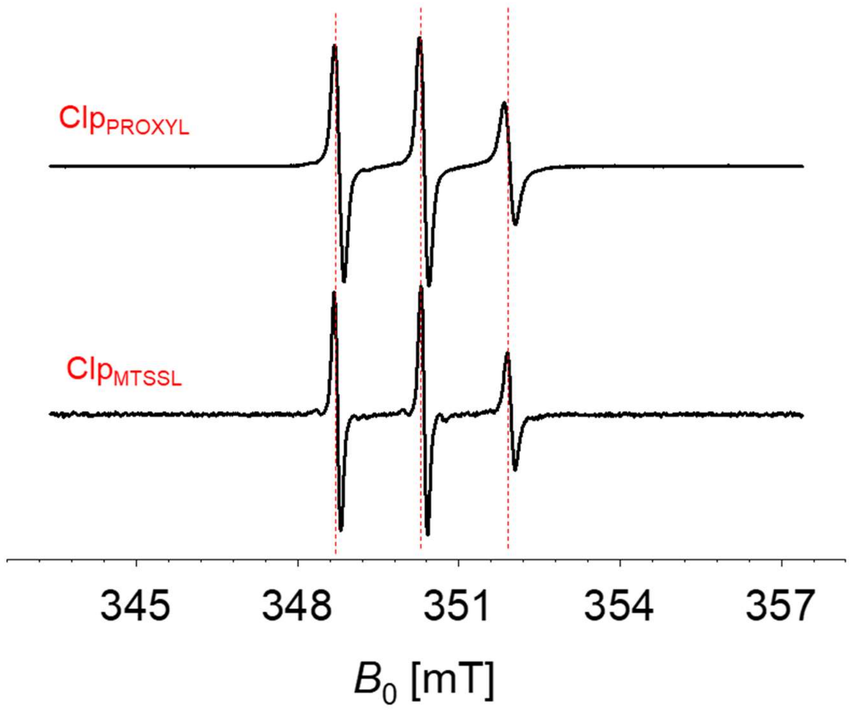

Supplementary Figure 9

CW EPR spectra for MTSSL-labeled coilin peptide (clp-MTSSL) and PROXYL-labeled coilin peptide (clp-PROXYL). Spectra recorded at room temperature at X-band frequency (9.76 GHz). Both systems exhibit correlation times of the spin label in the fast-regime (*f*). Source data are provided as a Source Data file.

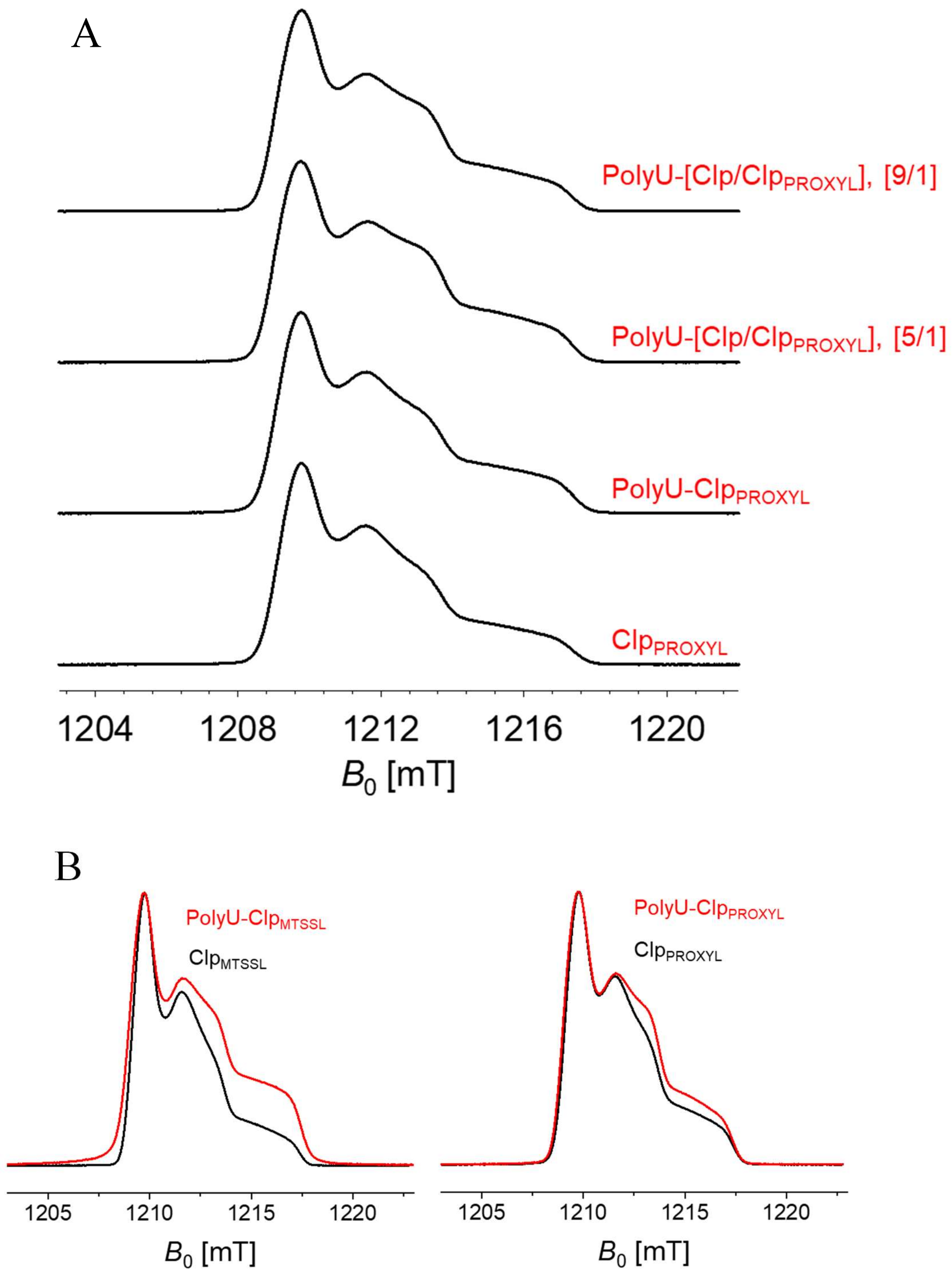

Supplementary Figure 10

Echo-Detected Field-Swept (EDFS) spectra for PROXYL-labeled coilin peptide (clp-PROXYL) upon addition of PolyU and unlabeled coilin peptide. (**A**) Spectra recorded at 50K at Q-band frequency (34 GHz). (**B**) Overlapping for the EDFS containing two different labels (MTSSL and PROXYL). Source data are provided as a Source Data file.

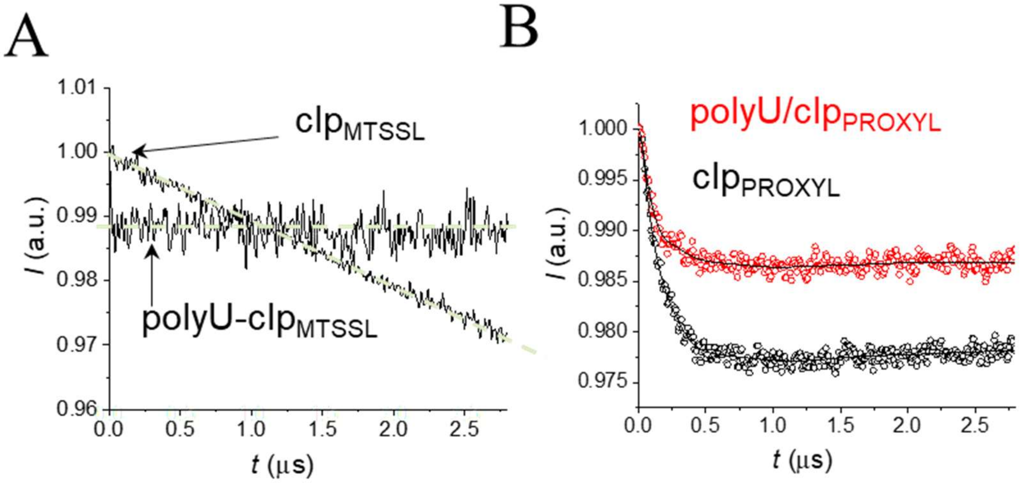

Supplementary Figure 11

Pulsed-EPR analysis of the MTSSL-labeled and PROXYL-labeled coilin peptide (clp$_{MTSSL}$ and clp$_{PROXYL}$) before and after polyU RNA-induced LLPS. (**A**) Dipolar evolution function without background corrections for the PELDOR experiment on polyU/clp mixture containing the MTSSL probes exhibiting clear difference on the *slope* of the PELDOR signal. (**B**) Dipolar evolution function with background corrections for the PELDOR experiment on polyU/clp mixture containing the PROXYL probes; in this case difference in modulation depths (λ) has been observed. Source data are provided as a Source Data file.

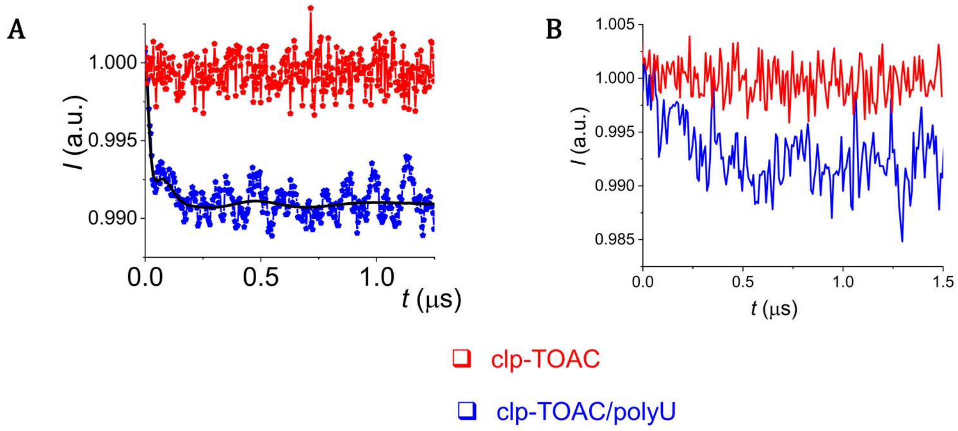

Supplementary Figure 12

Dipolar evolution functions (background corrected) for the clp (red) and clp/polyU (blue) samples (**A**, *left*); another set of DEER/PELDOR experiments recorded on a second set of samples of clp and clp/polyU, in order to show a reproducible effect (**B**, *right*) for samples containing TOAC probes. Source data are provided as a Source Data file.

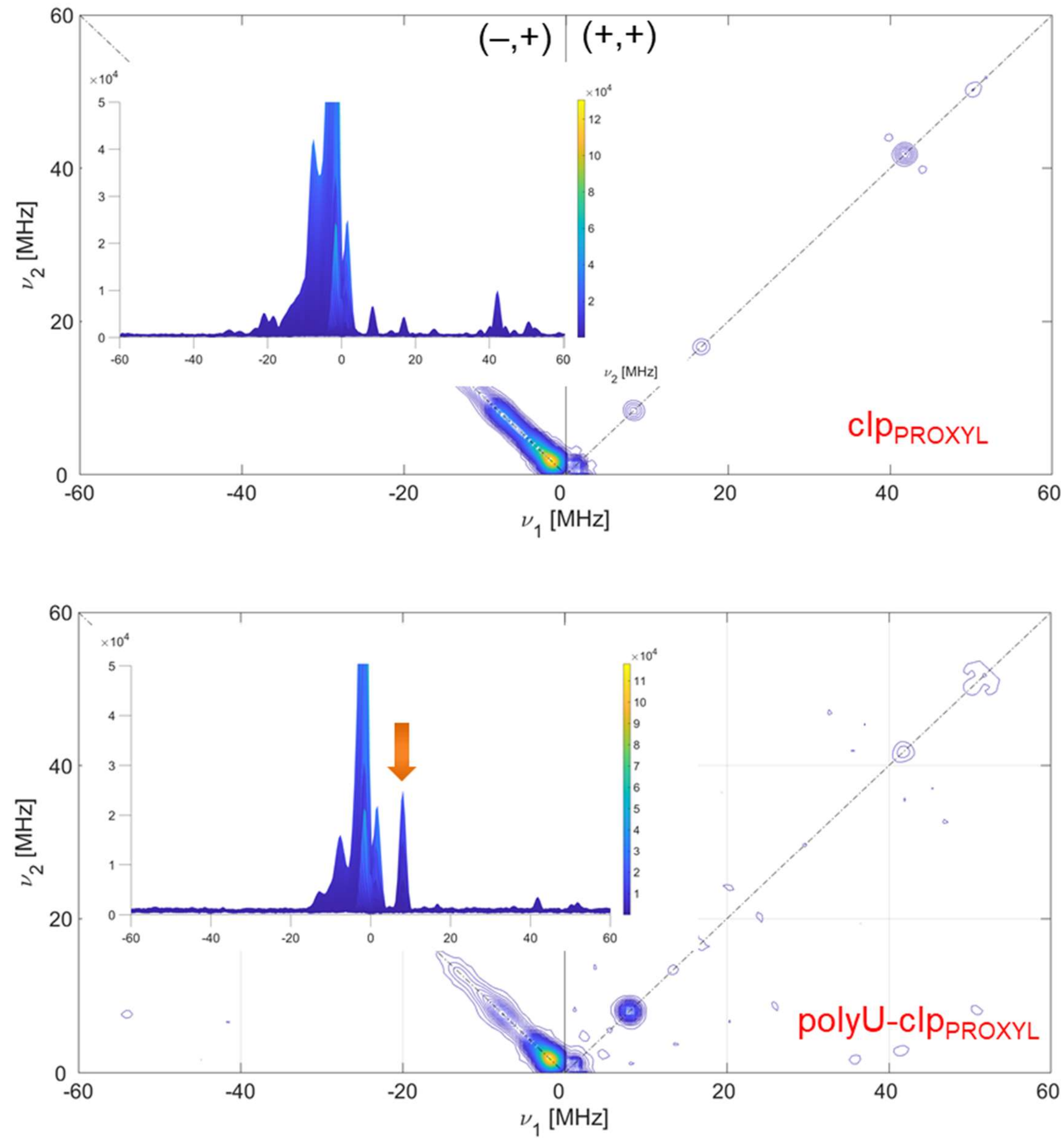

Supplementary Figure 13

Hyperfine sublevel correlation (HYSCORE) spectroscopy: 2D plot for the HYSCORE experiments on clp (*top*) and clp/polyU (*bottom*); (–, +) and (+,+) quadrants showing the diagonal and anti-diagonal peaks and cross-peaks generated by hyperfine interactions. For the (+,+) quadrant of the polyU/clp$_{PROXYL}$ sample; when clp undergoes LLPS, the ^{14}N signal is significantly increased with respect to ^{1}H and combination peaks.

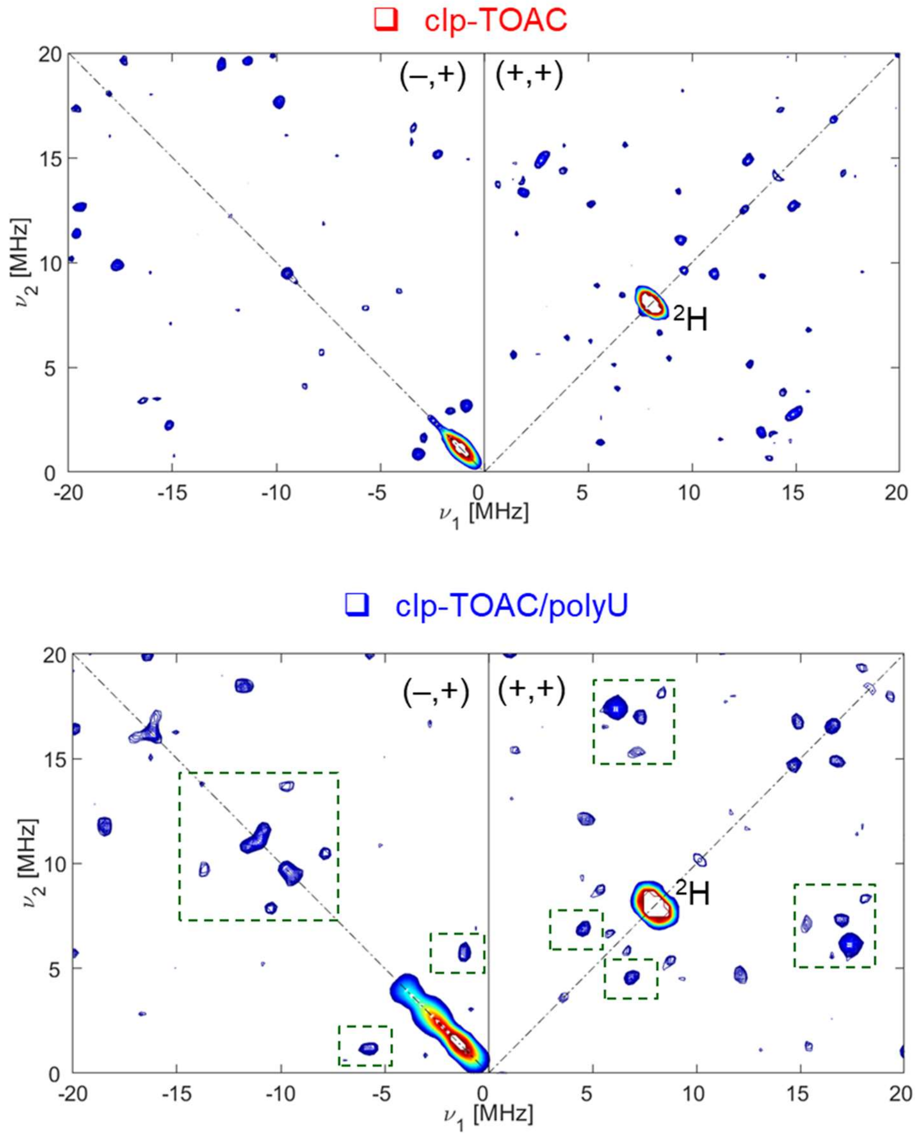

Supplementary Figure 14

Hyperfine sublevel correlation (HYSCORE) spectroscopy: 2D plot for the HYSCORE experiments on clp (*top*) and clp/polyU (*bottom*); (–, +) and (+,+) quadrants showing the diagonal and anti-diagonal peaks and cross-peaks generated by hyperfine interactions. For the (+,+) and (–, +) quadrants of the polyU/clp$_{\text{TOAC}}$ sample; in this case the S/N is low but additional peaks upon LLPS are still observable.

Supplementary Table 1

The parameters used in *gc*M2S* experiments, defined and calculated as described in ref. [1], then optimized for the experiment.

ε	$\Delta\nu$/Hz	J/Hz	n_1	n_2	τ/ms	Δ/ms
0.7	11.8	17	1	1	24	18
1.3	22	17	1	0	17.9	14.9
1.7	29.1	17	1	0	14.8	13.7
2.7	46	17	2	1	10.1	6.6

*. The *gc*M2S acronym stands for "general coupling Magnetization-to-Singlet", referring to a recently developed pulse sequence cited above. This pulse sequence allows accessing singlet-states in spin-pair systems coupled over a broad range of coupling regimes.

Supplementary Table 2

The principal components of *g*-factor and hyperfine (*A*) and nuclear quadrupole (*Q*) coupling tensors obtained from HYSCORE fit (Hyscorean software supported on MatLab package, version R2020b). The errors represent standard errors of fitting parameters.

g_x	2.00905 ± 0.00005
g_y	2.00693 ± 0.00005
g_z	2.00256 ± 0.00005
A_x (MHz)	5.30 ± 0.05
A_y (MHz)	2.95 ± 0.05
A_z (MHz)	5.60 ± 0.05
Q_x (MHz)	1.68 ± 0.05
Q_y (MHz)	2.10 ± 0.05
Q_z (MHz)	0.42 ± 0.05

Supplementary References

1. Mamone S, Rezaei-Ghaleh N, Opazo F, Griesinger C, Gloggler S. Singlet-filtered NMR spectroscopy. *Sci. Adv.* **6**, eaaz1955 (2020).
