# Supplementary material for: Large dynamics of a phase separating arginine-glycine-rich domain revealed via nuclear and electron spins
Source: Nat Commun. 2024 Feb 21;15:1610. doi: 10.1038/s41467-024-45788-w (PMC10881997; doi:10.1038/s41467-024-45788-w)
Supplement: Supplementary file 3 — Description of Additional Supplementary Files [file 41467_2024_45788_MOESM3_ESM.pdf]

Description of Additional Supplementary Files

Supplementary Data

Chromatograms and mass spectra of the two in-house synthesized peptides: coilin peptide without (Clp) or with an additional cysteine attached to the N-terminus (Cys-clp).

Supplementary Movie Legends

Supplementary Movie 1: Brownian motion of polyU-clp droplets captured via confocal fluorescence microscopy, using SYBR Gold as the fluorescent dye. The formation of relatively persistent droplet “twins” is illustrated by following the movement of selected droplets with time using the TrackMate ImageJ plugin.

Supplementary Movie 2: Brownian motion of polyU-clp droplets captured via confocal fluorescence microscopy, using SYBR Gold as the fluorescent dye. The inability of droplet “twins” to fuse with one another is illustrated here.
