# Supplementary material for: Large dynamics of a phase separating arginine-glycine-rich domain revealed via nuclear and electron spins
Source: Nat Commun. 2024 Feb 21;15:1610. doi: 10.1038/s41467-024-45788-w (PMC10881997; doi:10.1038/s41467-024-45788-w)
Supplement: Supplementary file 4 — Supplementary Data [file 41467_2024_45788_MOESM4_ESM.pdf]

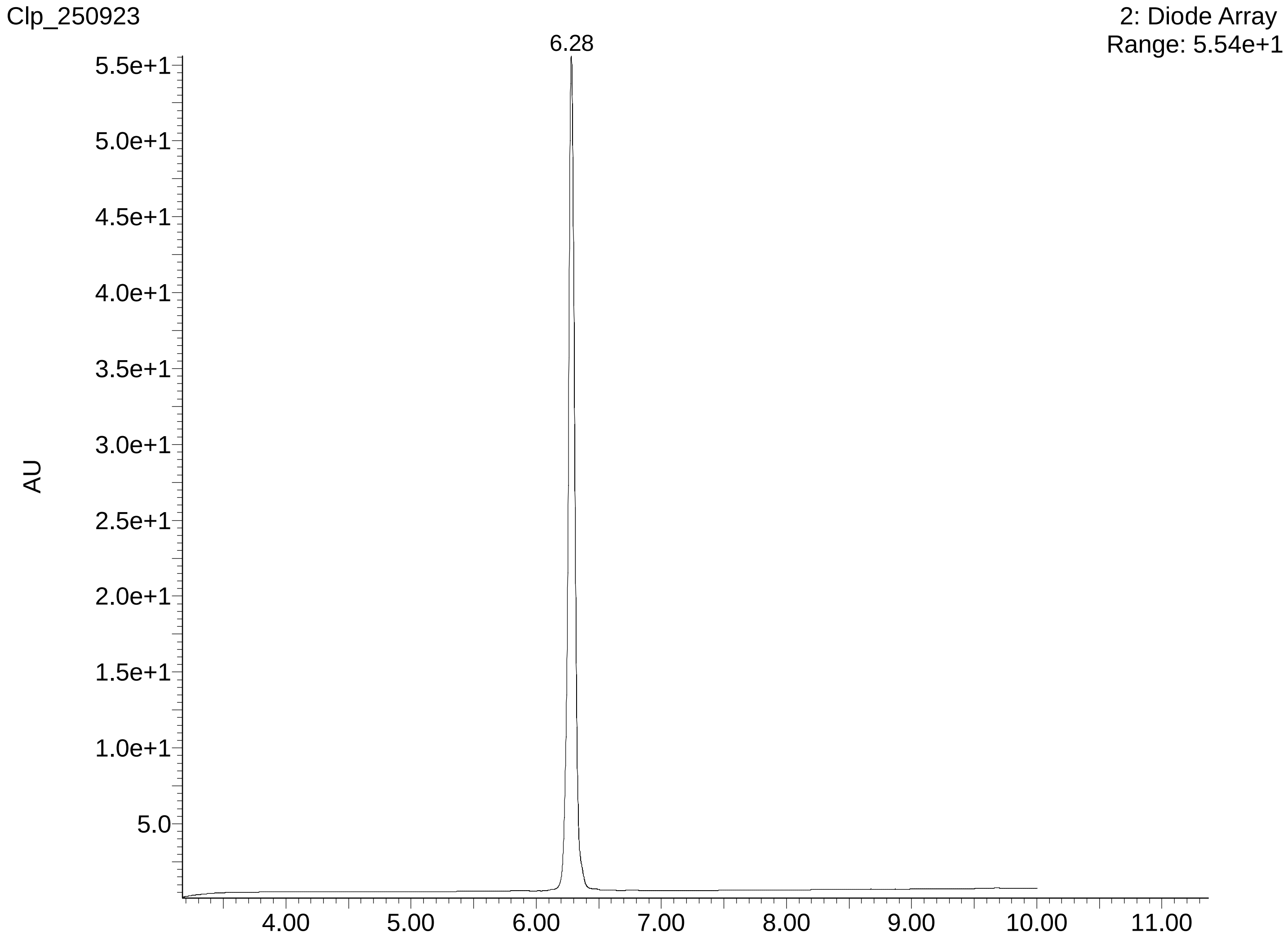
Clp_250923
2: Diode Array
Range: 5.54e+1
6.28
AU
5.5e+1
5.0e+1
4.5e+1
4.0e+1
3.5e+1
3.0e+1
2.5e+1
2.0e+1
1.5e+1
1.0e+1
5.0
4.00
5.00
6.00
7.00
8.00
9.00
10.00
11.00

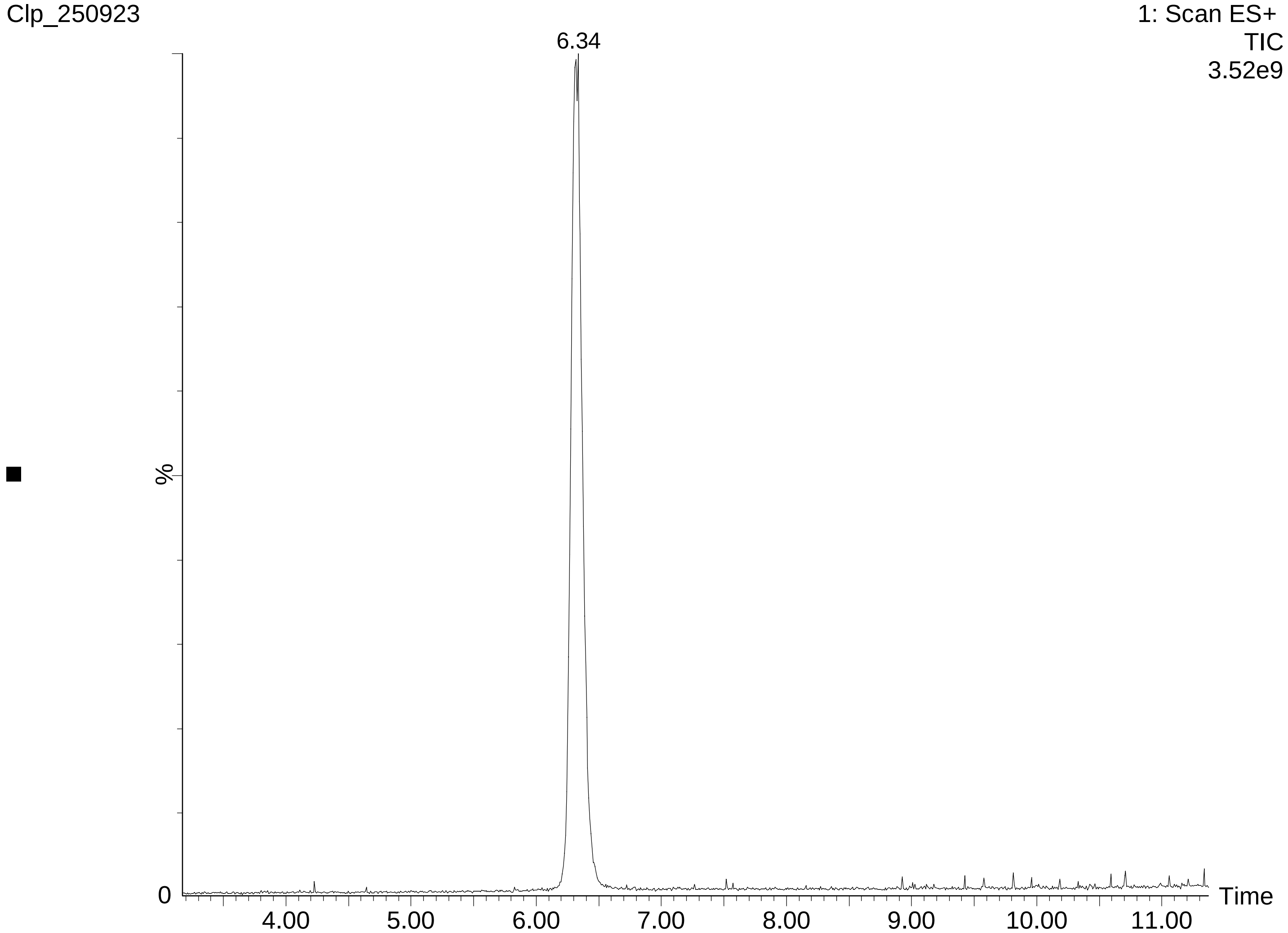
Clp_250923
1: Scan ES+
TIC
3.52e9
6.34
%
0
Time
4.00
5.00
6.00
7.00
8.00
9.00
10.00
11.00

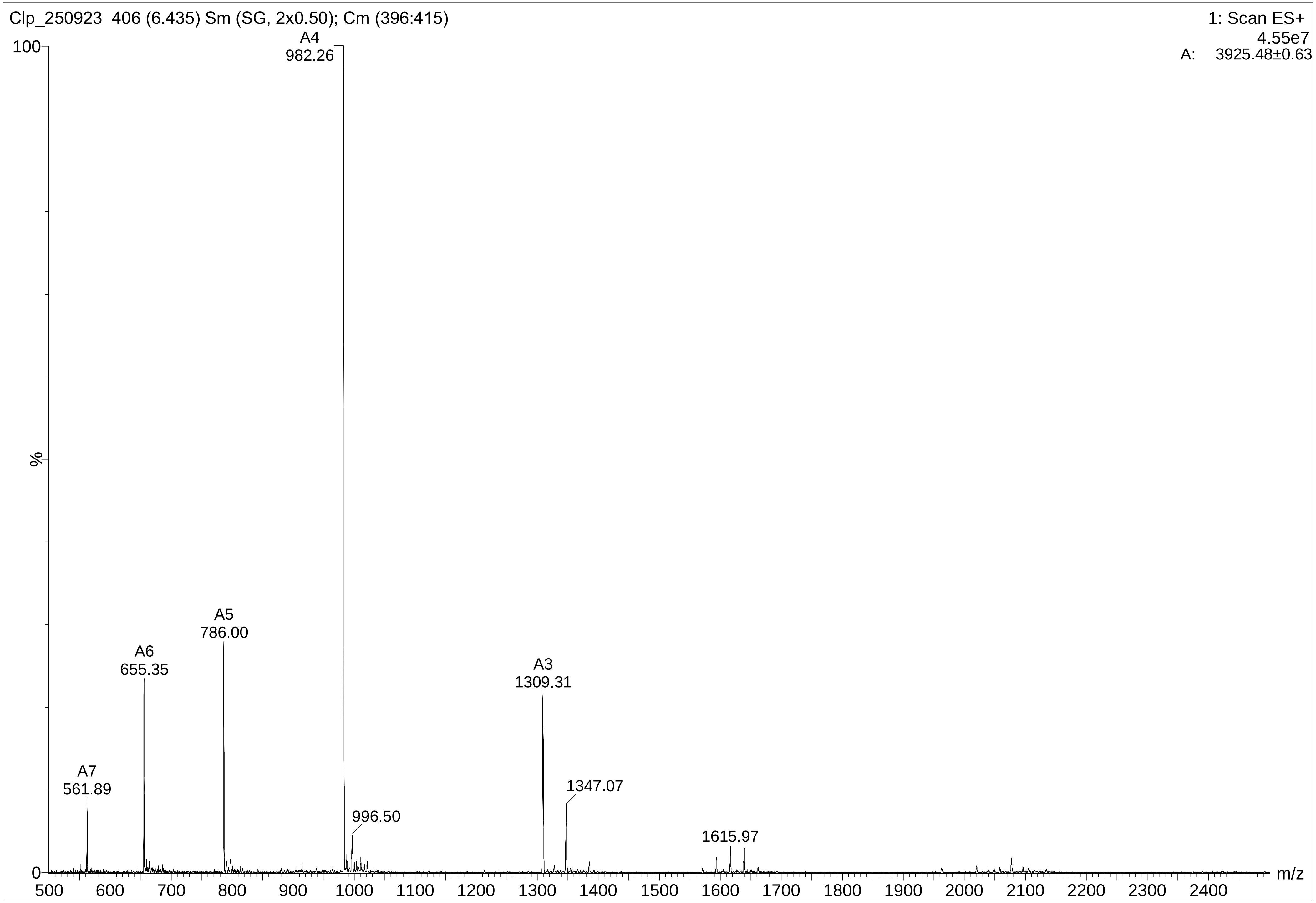

Clp_250923 406 (6.435) Sm (SG, 2x0.50); Cm (396:415)
1: Scan ES+
4.55e7
A: 3925.48±0.63
100
%
0
A4
982.26
A5
786.00
A6
655.35
A7
561.89
A3
1309.31
1347.07
996.50
1615.97
500
600
700
800
900
1000
1100
1200
1300
1400
1500
1600
1700
1800
1900
2000
2100
2200
2300
2400
m/z

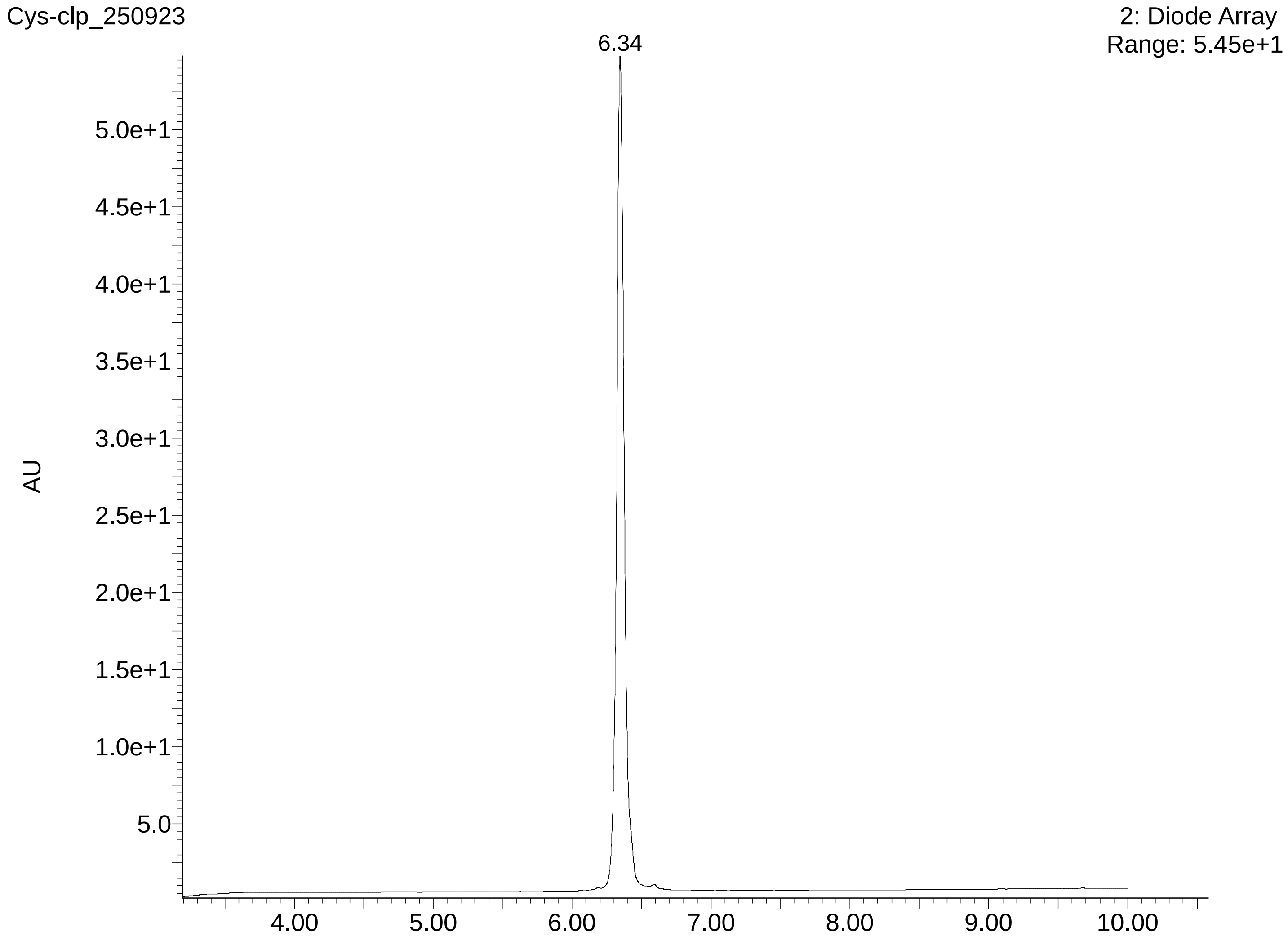
Cys-clp_250923
2: Diode Array
Range: 5.45e+1
6.34
5.0e+1
4.5e+1
4.0e+1
3.5e+1
3.0e+1
AU
2.5e+1
2.0e+1
1.5e+1
1.0e+1
5.0
4.00
5.00
6.00
7.00
8.00
9.00
10.00
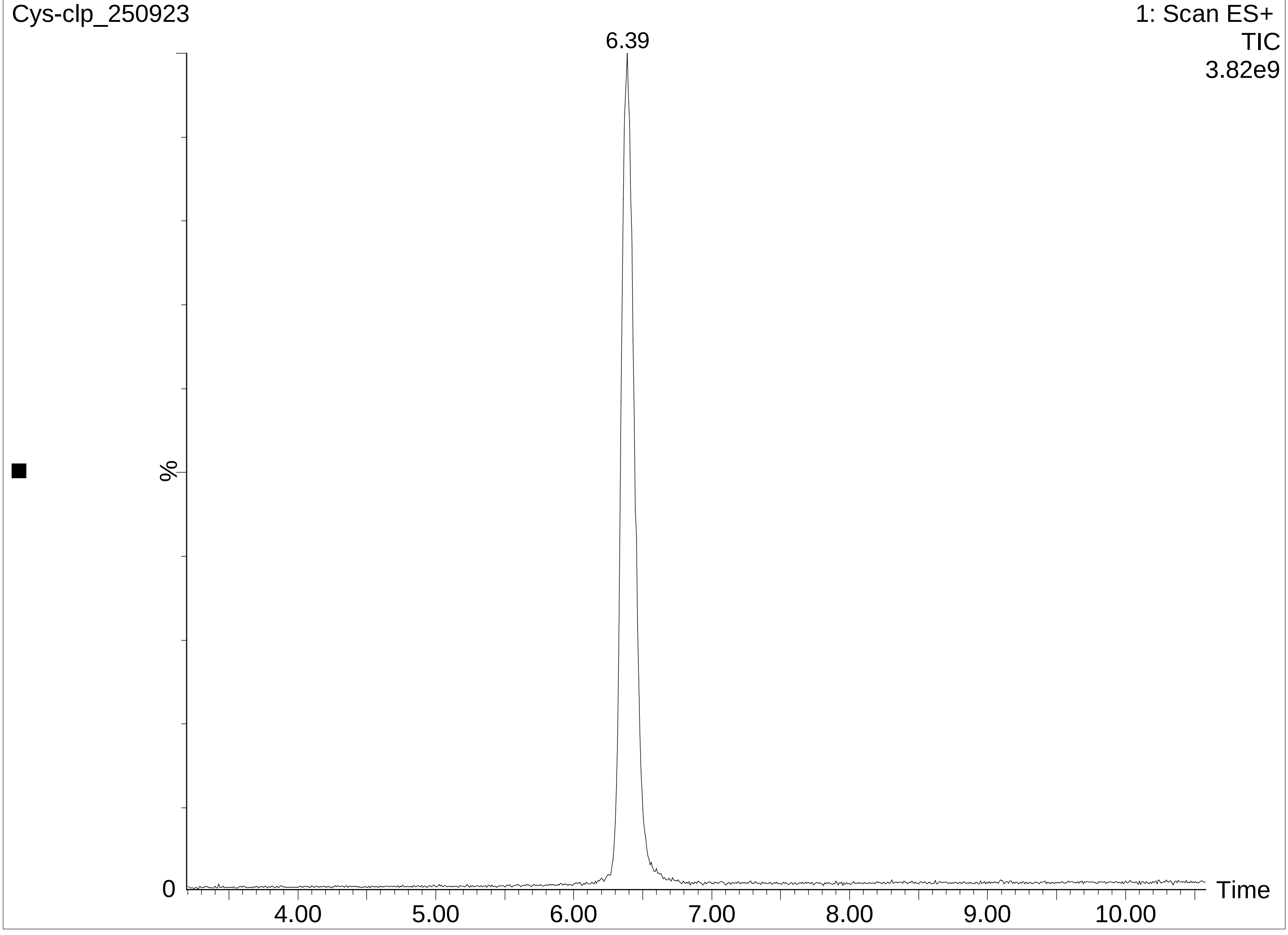
Cys-clp_250923
1: Scan ES+
TIC
3.82e9
6.39
%
0
Time
4.00
5.00
6.00
7.00
8.00
9.00
10.00

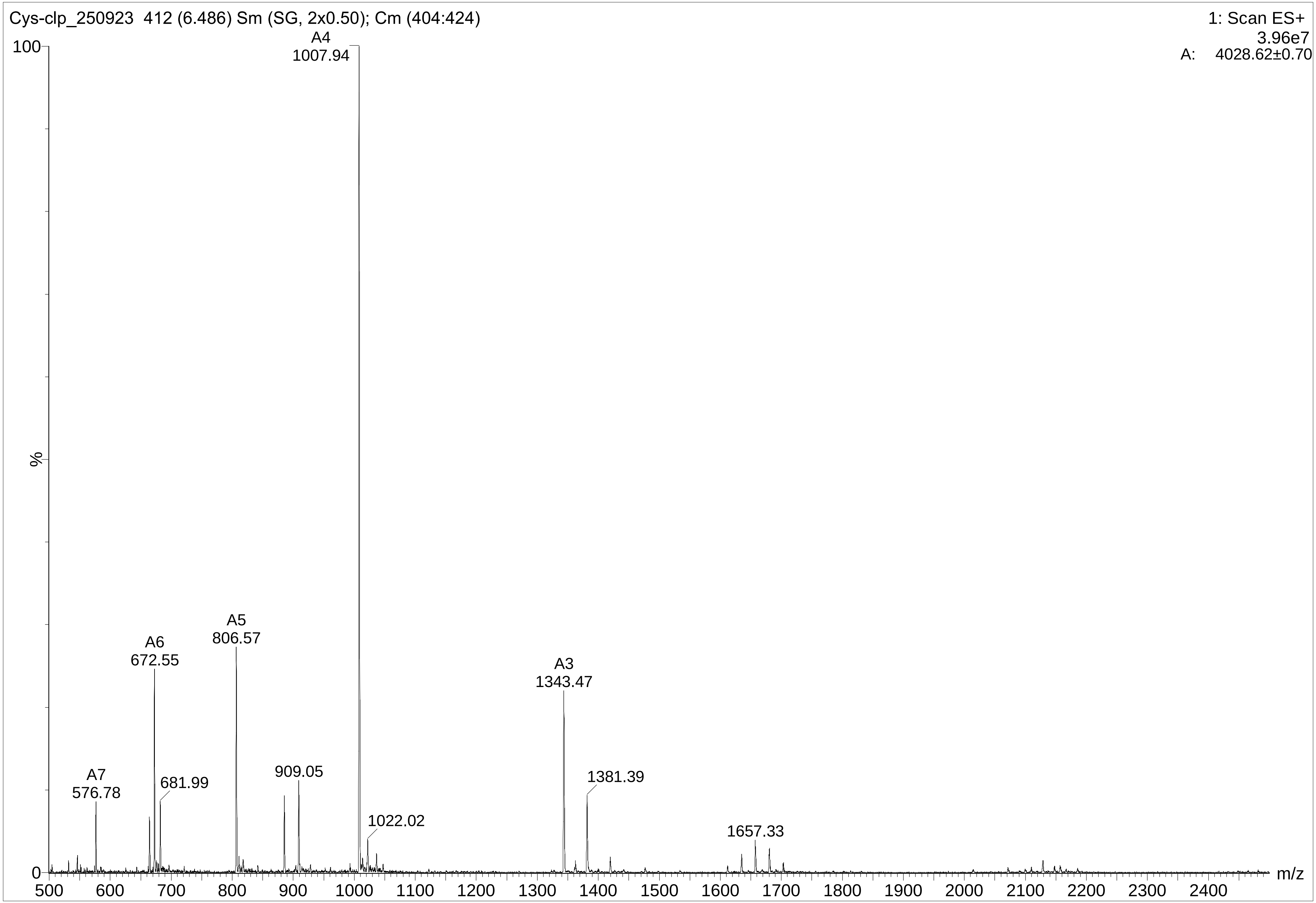
Cys-clp_250923 412 (6.486) Sm (SG, 2x0.50); Cm (404:424)
1: Scan ES+
3.96e7
A: 4028.62±0.70
A4
1007.94
A5
806.57
A6
672.55
A3
1343.47
A7
576.78
681.99
909.05
1381.39
1022.02
1657.33
%
100
0
m/z
500 600 700 800 900 1000 1100 1200 1300 1400 1500 1600 1700 1800 1900 2000 2100 2200 2300 2400
