# Supplementary material for: Large dynamics of a phase separating arginine-glycine-rich domain revealed via nuclear and electron spins
Source: Nat Commun. 2024 Feb 21;15:1610. doi: 10.1038/s41467-024-45788-w (PMC10881997; doi:10.1038/s41467-024-45788-w)
Supplement: Supplementary file 7 — Reporting Summary [file 41467_2024_45788_MOESM7_ESM.pdf]

nature portfolio

Corresponding author(s): Nasrollah Rezaei-Ghaleh

Last updated by author(s): Jan 25, 2024

Reporting Summary

Nature Portfolio wishes to improve the reproducibility of the work that we publish. This form provides structure for consistency and transparency in reporting. For further information on Nature Portfolio policies, see our Editorial Policies and the Editorial Policy Checklist.

Statistics

For all statistical analyses, confirm that the following items are present in the figure legend, table legend, main text, or Methods section.

n/a	Confirmed	
☐	☒	The exact sample size (*n*) for each experimental group/condition, given as a discrete number and unit of measurement
☐	☒	A statement on whether measurements were taken from distinct samples or whether the same sample was measured repeatedly
☒	☐	The statistical test(s) used AND whether they are one- or two-sided<br>*Only common tests should be described solely by name; describe more complex techniques in the Methods section.*
☒	☐	A description of all covariates tested
☒	☐	A description of any assumptions or corrections, such as tests of normality and adjustment for multiple comparisons
☒	☐	A full description of the statistical parameters including central tendency (e.g. means) or other basic estimates (e.g. regression coefficient) AND variation (e.g. standard deviation) or associated estimates of uncertainty (e.g. confidence intervals)
☒	☐	For null hypothesis testing, the test statistic (e.g. *F*, *t*, *r*) with confidence intervals, effect sizes, degrees of freedom and *P* value noted<br>*Give P values as exact values whenever suitable.*
☒	☐	For Bayesian analysis, information on the choice of priors and Markov chain Monte Carlo settings
☒	☐	For hierarchical and complex designs, identification of the appropriate level for tests and full reporting of outcomes
☒	☐	Estimates of effect sizes (e.g. Cohen's *d*, Pearson's *r*), indicating how they were calculated

Our web collection on statistics for biologists contains articles on many of the points above.

Software and code

Policy information about availability of computer code

Data collection	The NMR and EPR experiments were conducted using standard Bruker or previously published pulse sequences, as cited in the paper.
Data analysis	NMR and EPR data collection and analyses were done using Bruker Topspin version 3.x or 4.x and programs POKY (Build 20220825), TALOS-N (version 4.12 Rev 2015.147.15.40), easyspin (version 5.2.35) and MatLab (R2020b). The open source platform FIJI was used for microscope image analysis. The TrackMate 7 Image J plugin was used to follow the movement of selected droplets in fluorescence microscopy videos.

For manuscripts utilizing custom algorithms or software that are central to the research but not yet described in published literature, software must be made available to editors and reviewers. We strongly encourage code deposition in a community repository (e.g. GitHub). See the Nature Portfolio guidelines for submitting code & software for further information.

Data

Policy information about availability of data

All manuscripts must include a data availability statement. This statement should provide the following information, where applicable:

- Accession codes, unique identifiers, or web links for publicly available datasets
- A description of any restrictions on data availability
- For clinical datasets or third party data, please ensure that the statement adheres to our policy

Source data are provided with this paper. The sequence of protein coilin was taken from the UniProt entry with the code, P38432.

Research involving human participants, their data, or biological material

Policy information about studies with human participants or human data. See also policy information about sex, gender (identity/presentation), and sexual orientation and race, ethnicity and racism.

Reporting on sex and gender	N/A
Reporting on race, ethnicity, or other socially relevant groupings	N/A
Population characteristics	N/A
Recruitment	N/A
Ethics oversight	N/A

Note that full information on the approval of the study protocol must also be provided in the manuscript.

Field-specific reporting

Please select the one below that is the best fit for your research. If you are not sure, read the appropriate sections before making your selection.

☒ Life sciences ☐ Behavioural & social sciences ☐ Ecological, evolutionary & environmental sciences

For a reference copy of the document with all sections, see nature.com/documents/nr-reporting-summary-flat.pdf

Life sciences study design

All studies must disclose on these points even when the disclosure is negative.

Sample size	In analysis of fluorescence intensity ratio shown in Supplementary Figure 1, we used, as stated in Methods, an unbiased method of threshold setting, then applied a parameterized algorithm for particle search and ended up with the given number of droplets (65 for coilin and 85 for polyU RNA), which were all included in our analysis.
Data exclusions	None.
Replication	Samples were prepared for all the experiments (microscopy and NMR and EPR spectroscopy), as many times as needed. The reproducibility of phase separation was confirmed by inspection and microscopy and spectroscopy data. All the instances of repeated experiments were successful.
Randomization	We included all the microscopy and spectroscopic measurements in our analyses, the randomization of data was not required.
Blinding	The present study did not involve any clinical trial, animal study, immunoassay or similar experiments. The blinding (single, double) approaches were not applicable to this study.

Reporting for specific materials, systems and methods

We require information from authors about some types of materials, experimental systems and methods used in many studies. Here, indicate whether each material, system or method listed is relevant to your study. If you are not sure if a list item applies to your research, read the appropriate section before selecting a response.

Materials & experimental systems

n/a	Involved in the study	
☒	☐	Antibodies
☒	☐	Eukaryotic cell lines
☒	☐	Palaeontology and archaeology
☒	☐	Animals and other organisms
☒	☐	Clinical data
☒	☐	Dual use research of concern
☒	☐	Plants

Methods

n/a	Involved in the study	
☒	☐	ChIP-seq
☒	☐	Flow cytometry
☒	☐	MRI-based neuroimaging
